# Detection of Epileptic Seizure Event and Onset Using EEG

**DOI:** 10.1155/2014/450573

**Published:** 2014-01-29

**Authors:** Nabeel Ahammad, Thasneem Fathima, Paul Joseph

**Affiliations:** Department of Electrical Engineering, National Institute of Technology, Calicut, Kerala 673601, India

## Abstract

This study proposes a method of automatic detection of epileptic seizure event and onset using wavelet based features and certain statistical features without wavelet decomposition. Normal and epileptic EEG signals were classified using linear classifier. For seizure event detection, Bonn University EEG database has been used. Three types of EEG signals (EEG signal recorded from healthy volunteer with eye open, epilepsy patients in the epileptogenic zone during a seizure-free interval, and epilepsy patients during epileptic seizures) were classified. Important features such as energy, entropy, standard deviation, maximum, minimum, and mean at different subbands were computed and classification was done using linear classifier. The performance of classifier was determined in terms of specificity, sensitivity, and accuracy. The overall accuracy was 84.2%. In the case of seizure onset detection, the database used is CHB-MIT scalp EEG database. Along with wavelet based features, interquartile range (IQR) and mean absolute deviation (MAD) without wavelet decomposition were extracted. Latency was used to study the performance of seizure onset detection. Classifier gave a sensitivity of 98.5% with an average latency of 1.76 seconds.

## 1. Introduction

Epilepsy is one of the most prevalent neurological disorders in human beings. It is characterized by recurring seizures in which abnormal electrical activity in the brain causes the loss of consciousness or a whole body convulsion. Patients are often unaware of the occurrence of seizure due to the random nature of them which may increase the risk of physical injury. Studies show that 4-5% of the total world population has been suffering from epilepsy [1].

Electroencephalogram is one of the important tools for diagnosis and analysis of epilepsy. Electroencephalogram is the recorded representation of electrical activity produced by firing of neuron within the brain along the scalp. For recording of EEG, electrodes will be pasted at some key points on the patient's head. Electrodes pick up the signals and will be recorded in a recording device through wires which are connected to electrodes. The “10-20” system is the internationally recognized method to apply the location of electrodes in EEG recording. The “10-20” refers to the fact that actual distances between electrodes are either 10% or 20% of front-back or right-left distance of the skull [2–4].

As complete visual analysis of EEG signal is very difficult, automatic detection is preferred. Fourier transform has been most commonly used in early days of processing of EEG signals. However as EEG signal is a nonstationary signal, Fourier analysis does not give accurate results [5–7]. Most effective time-frequency analysis tool for analysis of transient signal is wavelet transform [8–10].

The automated diagnosis of epilepsy can be subdivided into preprocessing, feature extraction, and classification. Seizure detection can be classified as either seizure onset detection or seizure event detection. In seizure onset detection the purpose is to recognize the starting of seizure with the shortest possible delay. The purpose of seizure event detection is to identify seizures with the highest possible accuracy [11–16].

For treatment of epilepsy, patients take antiepileptic drugs on daily basis. But about 25% of them again experience frequent seizures. For these patients, surgery is the most important and generally adopted treatment method. Surgery can be done only if epileptogenic focus is identified accurately. For this purpose different types of tracers are used as soon as seizure onset is detected. Hence the seizure onset detection is very important [1].

Seizure detection from EEG signal was started since 1980s. In 1982 Gotman proposed a remarkable work on seizure detection [5]. Khan and Gotman proposed a wavelet based method for classification of epileptic and nonepileptic data [17]. In 2005 wavelet transform method and short time Fourier transform method were compared to determine their accuracy to determine the epileptic seizures. They found that wavelet transform method gives better performance [18]. Übeyli suggested the combined neural network model for the classification using wavelet based features [12]. Their method gave good accuracy in Bonn University data. In 2011, Gandhi et al. made a comparative study of wavelet families for EEG signal classification [11]. Important features such as energy, entropy, and standard deviation at different subbands were computed using wavelet decomposition. Feature vector was used to model and train the probabilistic neural network and classification accuracies were evaluated for each of the wavelet families. The result obtained was compared with support vector machine classifier.

An onset detection system was designed by Gotman and Saab in 2004. They achieved a median detection delay of 9.8 sec and sensitivity of 77.9% using scalp EEG. Shoeb and Guttag achieved 96% sensitivity and small detection delays [6]. Sorensen et al. achieved 78–100% sensitivity when using a matching pursuit algorithm and with 5–18 seconds delay in seizure onset detection [19].

The outline of this study is as follows.  Section 2 explains about the materials and methods used in this study. It includes wavelet transforms used for EEG signal processing, parameters used for classification, linear classifier, terms used to describe the performance of the classifier, and description about databases.  Section 3 includes results and discussions, and the last section gives the conclusion.

## 2. Materials and Methods

### 2.1. Wavelet Transform

Wavelet transform is the representation of a time function in terms of simple, fixed building blocks termed as wavelets. These building blocks are a family of functions which are derived from a single generating function called mother wavelets using translation and dilation operations. The main advantage of wavelet transform is that it has varying window size, being broad at low frequency and narrow at high frequency. It leads to an optimal time-frequency resolution in all frequency ranges. By performing spectral analysis using wavelet transform, EEG signals consisting of many data points can be compressed into few features [20–23].

**Figure 1:**
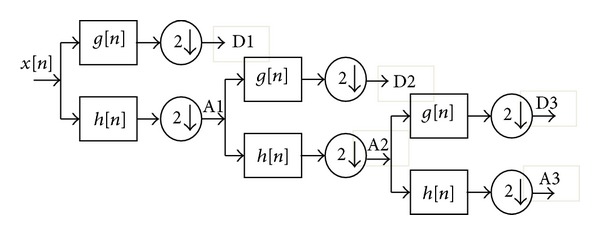
Subband decomposition of discrete wavelet transform implementation.

The wavelet transform can be categorized into continuous and discrete types. Continuous wavelet transform is defined as

(1) $$\begin{array}{c} \text{CWT}\left(a,b\right)=\int_{-\infty }^{\infty }x\left(t\right)\Psi_{a,b}^{*}\left(t\right)dt, \end{array}$$

where *x*(*t*) represents the analyzed signal and *a* and *b* represent the scaling factor (dilation/compression coefficient) and translation along time axis (shifting coefficient), respectively. The superscript asterisk denotes the complex conjugation. Ψ_*a*,*b*_(·) is obtained by scaling the wavelet at time *b* and scale *a*:

(2) $$\begin{array}{c} \Psi_{a,b}^{}\left(t\right)=\frac{1}{\sqrt{\left|a\right|}}\Psi \left(\frac{t-b}{a}\right), \end{array}$$

where Ψ(*t*) represents the wavelet. In continuous WT, the scaling and translation parameters “*a*” and “*b*” change continuously. However, calculating wavelet coefficients for every possible scale can represent a considerable effort and result in a vast amount of data. Therefore discrete wavelet transform is commonly used. The wavelet transform can be thought of as an extension of classic Fourier transform. Instead of working on a single scale (time or frequency), it works on a multiscale basis. Multiresolution decomposition of a signal *x*[*n*] is schematically shown in Figure 1.

Each stage of this scheme consists of two digital filters and two down samplers. The first filter *g*[·] is high pass in nature and the second *h*[·] is its mirror version which is low pass in nature. The downsampled outputs of first high-pass and low-pass filters provide the detail D1 and approximation A1, respectively. The first approximation A1 is further decomposed and this process is continued up to the level we required as shown in Figure 1.

### 2.2. Parameters for Feature Extraction

The EEG signals, which contain many data points, can be compressed into few features that can discriminate between different classes. The features used include some wavelet based features and some statistical features without wavelet decomposition.

(*1) Wavelet Based Features.* Energy, entropy, standard deviation, mean, maximum, and minimum were used as parameters after wavelet decomposition.

The energy at each decomposition level was calculated as

(3) $$\begin{array}{c} {\text{ED}}_{i}=\sum_{j=1}^{N}{\left|D_{ij}\right|}^{2},\;i=1,2,\ldots ,l,\\ {\text{EA}}_{i}=\sum_{j=1}^{N}{\left|A_{ij}\right|}^{2}. \end{array}$$

The entropy at each decomposition level was calculated as

(4) $$\begin{array}{c} {\text{ENT}}_{i}=\sum_{j=1}^{N}D_{ij}^{2}\log(D_{ij}^{2}),\;i=1,2,\ldots ,l, \end{array}$$

where *i* = 1,2,…, *l* is wavelet decomposition level from 1 to *l* and *N* is the number of coefficients of detail or approximation at each decomposition level.

The standard deviation at each decomposition level was calculated using the following equation:

(5) $$\begin{array}{c} \sigma_{i}={\left(\frac{1}{N-1}\sum_{j=1}^{N}{{(D}_{ij}-\mu_{i})}^{2}\right)}^{1/2}, \end{array}$$

where *μ*_*i*_ is the mean and is given by

(6) $$\begin{array}{c} \mu_{i}=\frac{1}{N}\sum_{j=1}^{N}D_{ij},\;i=1,2,\ldots ,l. \end{array}$$

(*2) Statistical Features without Wavelet Decomposition.* In latency study of seizure detection two statistical features: IQR and MAD, were computed over raw data. IQR (interquartile range) is a measure of statistical dispersion. It is the difference between upper quartile (*Q*_3_) and lower quartile (*Q*_1_):

(7) $$\begin{array}{c} \text{IQR}=Q_{3}-Q_{1}. \end{array}$$

MAD (mean absolute deviation) is the mean of absolute deviation from mean.

### 2.3. Classifier

The extracted features should be distinguished between normal and deviating cases. In classification stage all the features will be given to a classifier. In seizure detection problem this step is the classification between normal and epileptic EEG. In the present study linear classifier is used for classification.

In a linear classifier the classification is achieved by making a decision based on the value of linear combination of features. If the input features to the classifier are a real vector $\vec{x}$ then the output score is

(8) $$\begin{array}{c} y=f\left(\vec{w}\cdot \vec{x}\right)=f\left(\sum_{j}w_{j}x_{j}\right), \end{array}$$

where $\vec{w}$ is a real vector of weights and *f* is a function that converts the dot product of two vectors into the desired output. The weight vector is computed using a set of labeled training samples. Often *f* is simple function that maps all the values above a certain threshold to the first class and all other values to the second class. A more complex *f* might give the probability that items belong to certain class. A linear classifier is often used where speed of classification is an issue [9].

(*1) Performance of Classifier in Seizure Event Detection.* Specificity, sensitivity, and accuracy are used for determining the performance of classifiers. They are defined as

(9) $$\begin{array}{c} \text{specificity = }\frac{\text{number of true negative decisions}}{\text{number of actually negative cases}},\\ \text{sensitivity = }\frac{\text{number of true positive decisions}}{\text{number of actually positive cases}},\\ \text{accuracy = }\;\frac{\text{number of correct decisions}}{\text{total number of cases}}\text{.} \end{array}$$

(*2) Performance of Classifier in Seizure Onset Detection.* In seizure onset detection, performance of the detector is described by latency and sensitivity. Latency is the delay between the actual seizure onset and onset detected by the detector. If the value of latency is close to 0 the detector will have a good performance and if its value is far from 0 it will have poor performance.

### 2.4. EEG Data Sets Used

(*1) Data Set for Seizure Event Detection.* Bonn University data is used for the study of seizure event detection. The recording was done using standard 10-20 electrode placement system. The complete data sets consist of five sets each containing 100 channels which is named from A to E. Sets A and B consist of EEG segments taken from surface EEG recording carried out on five healthy volunteers. Volunteers were relaxed in an awaken state with eyes open (A) and eyes closed (B), respectively. Sets C, D, and E were taken from EEG archive of presurgical diagnosis. Segments in set D were recorded from the epileptogenic zone. Set C is recorded from hippocampal formation of opposite hemisphere of brain. Sets C and D contain only activity measured during seizure-free intervals. Set E contains only seizure activity [24].

Data is recorded within 128-channel amplifier system and digitized at 173.61 Hz sampling rate and 12 bit A/D resolution. To select the EEG signal of desired band a band-pass filter having a pass band of 0.53–40 Hz (12 dB/oct) was used. It was cut out from continuous multichannel EEG recordings after visual inspection for artifacts due to muscle activity or eye movement.

(*2) Data Set for Seizure Onset Detection.* CHB-MIT scalp EEG database is used for study of latency. It was collected from Boston Children's Hospital. The database consists of EEG recordings with intractable seizures recorded from pediatric subjects. Sampling rate of all signals is 256 samples per second with a resolution of 16 bit. For recording the international 10-20 system of EEG electrode positions and nomenclature were used [6].  Table 1 gives a brief overview of database used for latency study.

The EEG data set of each patient is segmented to records of typically one hour long. Records that contain seizure and that do not contain seizure are called seizure records and nonseizure records, respectively.

## 3. Results and Discussion

### 3.1. Seizure Event Detection

In the present study the data sets A, D, and E have been used. The data used has been already gone through the preprocessing steps. One channel consists of total 4096 samples. For one channel 16 rectangular windows were formed which consists of 256 discrete data.

(*1) Feature Extraction Using Discrete Wavelet Transform.* Selection of appropriate wavelet and the number of decomposition levels are very important in the analysis of signals using wavelet transform. The number of decomposition levels is chosen based on the dominant frequency components of the signal. The level of decomposition is chosen such that the frequencies required for classification of the signal are retained in the wavelet coefficients. In the present study the number of decomposition levels was chosen to be 4. Thus the EEG signals were decomposed into the details D1–D4 and one final approximation A4. The smoothening features of Daubechies wavelets of order 2 made it more suitable to detect changes of EEG signals [9]. Therefore, the wavelet coefficients were computed using Daubechies wavelets of order 2. The wavelets coefficients were computed using MATLAB software package.

**Figure 2:**
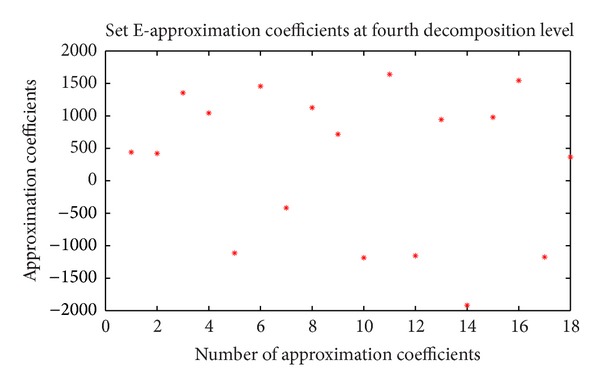
Approximation coefficient at fourth decomposition level of data set E (first frame of first channel).

**Figure 3:**
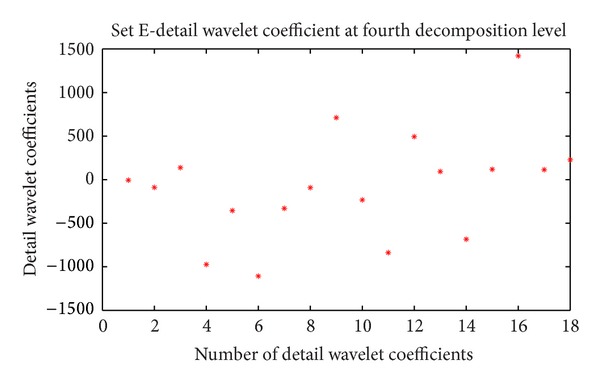
Detail wavelet coefficient at fourth decomposition level of data set E (first frame of first channel).

**Figure 4:**
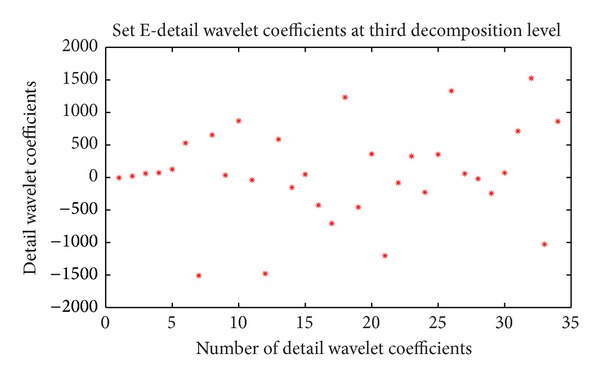
Detail wavelet coefficients at third decomposition level of data set E (first frame of first channel).

For each EEG segment, the detail wavelet coefficients at first, second, third, and fourth levels and approximation wavelet coefficients at fourth level were computed. In this study only D3, D4 and A4 are used because these coefficients represents the frequency ranges of interest [12]. The approximation coefficients at fourth level and detail wavelet coefficients at third and fourth levels of first frame of data set E are shown in Figures 2, 3, and 4, respectively.

Tables 2, 3, and 4 show the extracted features of first frames of data sets A, D, and E, respectively.

(*2) Classification Using Linear Classifier.* A linear classifier has been trained such that it gives an output of 0 for normal EEG, 1 for set E, and 2 for set D. The calculated features were given to this classifier. Out of total 16 frames 10 frames were used for training the classifier and the rest 6 frames were used for testing. Output of linear classifier has been shown in Table 5.

Table 6 shows the classification accuracies of linear classifier. Accuracy has been explained in terms of specificity, sensitivity, and total classification accuracy. Total classification accuracy achieved is 84.2%.

Some other researchers also worked on the same database. Übeyli [12] used wavelet based features along with a combination of neural network classifiers. Song and Liò [25] used sample entropy as feature and back propagation and extreme learning machine classifiers. Though the two works reported better accuracy in classification, these classifications are computationally rigorous. At the same time the present work uses the simple linear classifier.

### 3.2. Seizure Onset Detection

In CHB-MIT database the duration of each seizure is different. Each seizure was divided into frames of 1 second. Wavelet decomposition at four levels was done using Daubechies wavelet of order 2 for every frame. Six wavelet based features: maximum, minimum, mean, standard deviation, energy, and entropy, were computed for three wavelet coefficients, A4, D4, and D3 of last two levels of decomposition. Along with these features, two statistical features IQR and MAD were calculated for each channel of each frame without wavelet decomposition.

Hence there are total 20 features for each channel of each epoch. For each epoch a vector of 23∗20 dimension was formed because each epoch is having 23 channels. Since a seizure is having *T* such epochs a feature vector was formed by placing them vertically and forming a feature vector of (23∗*T*)∗20 dimension. This feature vector is for seizure EEG signal. In the same procedure as discussed above feature vector for normal EEG signal was calculated.

Classification was done using linear classifier to differentiate between seizure and normal EEG. Normal and seizure epochs were labeled using 0 and 1, respectively. Minimum of 60% of seizures were used for training and the remaining for testing for classification of each patient. Details about the number of seizures used for training and testing are described in Table 7. For example in the case of patient number 24, ten seizures were used for training and 6 for testing.

**Figure 5:**
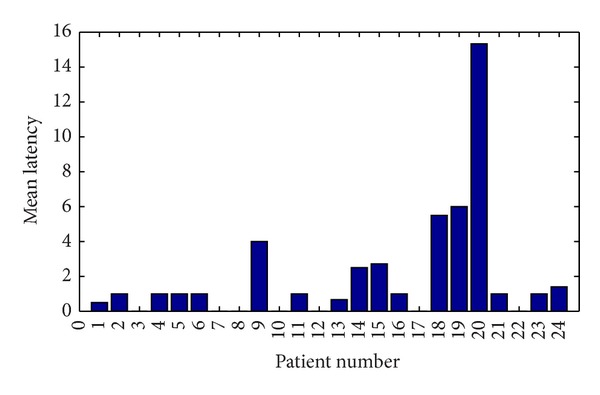
Mean latency of each patient.

**Figure 6:**
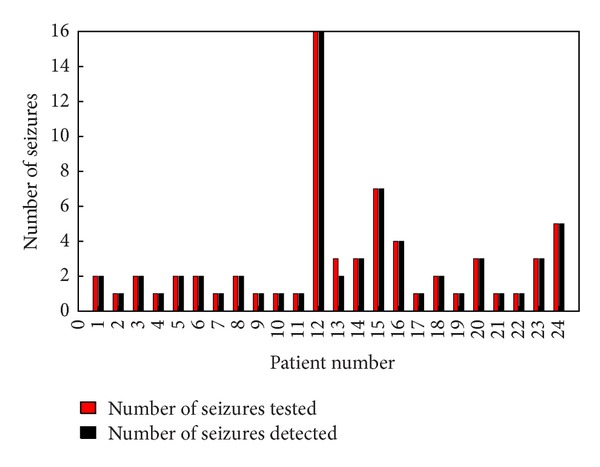
Sensitivity of the detector.

**Figure 7:**
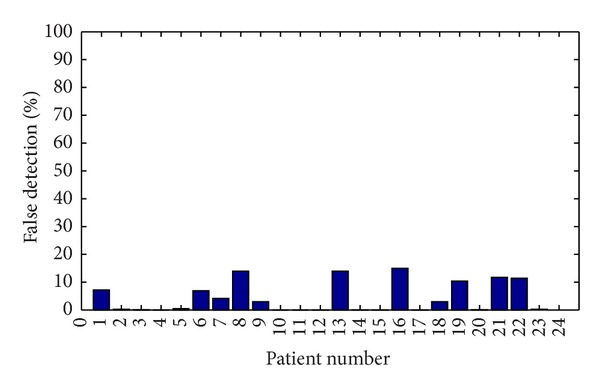
False detection percentage of each patient.

Classifier will declare a seizure in an epoch if at least 60% of channels show value of 1 as output. Latency and sensitivity were used to describe the performance of classifier.  Figure 5 shows the mean latency of each patient. From the graph it is clear that zero latency is achieved in the case of patients 3, 7, 8, 12, and 17. The average latency was found to be 1.76 seconds.  Figure 6 shows the sensitivity of the detector. All the seizures except one seizure in patient 14 have been detected. An average sensitivity of 98.5% has been achieved.  Figure 7 shows the false detection percentage which is near zero for 13 patients.

The comparison of results with that reported by Shoeb and Guttag [6] on the same database shows improvement in terms of sensitivity from 96% to 98.5% and average latency from 4.2 seconds to 1.76 seconds. Even though the false positive rate shows no improvement, the latency and sensitivity show much improvement.

## 4. Conclusion

In this work automatic detection methods of epileptic seizure event and onset have been proposed. In the case of seizure event detection Bonn University data has been decomposed with Daubechies wavelet of order 2 and six features such as maximum, minimum, mean, standard deviation, energy, and entropy were computed over the wavelet coefficients at third and fourth levels. Classification has been done using linear classifier and a total accuracy of 84.2% has been achieved.

In the case of seizure onset detection, CHB-MIT database has been used. Along with features used in seizure event detection, interquartile range and mean absolute deviation have been extracted. Latency and sensitivity are used to study the performance of the linear classifier. A sensitivity of 98.5% has been achieved with an average latency of 1.76 seconds.

## Figures and Tables

**Table 1:** An overview of CHB-MIT database.

Patient	Age	Gender	Number of seizures
1	11	F	7
2	11	M	3
3	14	F	6
4	22	M	4
5	7	F	5
6	1.5	F	3
7	14.5	F	3
8	3.5	M	5
9	10	F	4
10	3	M	4
11	12	F	3
12	2	F	40
13	3	F	11
14	9	F	8
15	16	M	18
16	7	F	10
17	12	F	3
18	18	F	6
19	19	F	3
20	6	F	8
21	13	F	4
22	9	F	3
23	6	F	7
24	—	—	16

**Table 2:** Extracted features of first frame of data set A.

Extracted features	D3	D4	A4
Maximum	75.7695	120.0146	192.677
Minimum	−92.3744	−105.366	−172.499
Mean	1.6022	2.1703	34.4130
Standard deviation	41.1865	60.3469	96.4623
Entropy	4.522*e* + 05	5.47*e* + 05	1.77*e* + 06
Energy	5.6*e* + 04	6.199*e* + 04	1.79*e* + 05

**Table 3:** Extracted features of first frame of data set D.

Extracted features	D3	D4	A4
Maximum	44.34	88.24	320.44
Minimum	−30.926	−89.15	−175.76
Mean	1.65	−2.63	94.15
Standard deviation	19.4	43.6354	126.3
Entropy	1.258*e* + 04	3.24*e* + 04	4.3*e* + 05
Energy	8.2*e* + 04	2.7*e* + 05	4.5*e* + 06

**Table 4:** Extracted features of first frame of data set E.

Extracted features	D3	D4	A4
Maximum	1524.4000	1420.100	1639.200
Minimum	−1508.9000	−117.0000	−1917.600
Mean	65.5614	−77.2298	281.4010
Standard deviation	716.0870	614.2615	1138.500
Entropy	2.38*e* + 08	8.9*e* + 07	2.39*e* + 08
Energy	1.7*e* + 07	6.9*e* + 06	2.39*e* + 08

**Table 5:** Confusion matrix of linear classifier output.

Testing set	Set A	Set D	Set E
Set A	514	86	0
Set D	135	456	9
Set E	9	45	456

**Table 6:** Classification accuracies.

Statistical parameters	Db2
Specificity	85.6%
Sensitivity (set D)	76%
Sensitivity (set E)	91%

Total classification accuracy	84.2%

**Table 7:** Number of seizures used for training and testing.

Patient number	Total number of seizures	Number of seizures used for training	Number of seizures used for testing
1	7	5	2
2	3	2	1
3	6	4	2
4	4	3	1
5	5	3	2
6	5	3	2
7	3	2	1
8	5	3	2
9	4	3	1
10	4	3	1
11	3	2	1
12	40	24	16
13	11	8	3
14	8	5	3
15	18	11	7
16	10	6	4
17	3	2	1
18	6	4	2
19	3	2	1
20	8	5	3
21	4	3	1
22	3	2	1
23	7	4	3
24	16	10	6
